# Lurasidone in adolescents with schizophrenia: Sustained remission and recovery during 2 years of open-label treatment

**DOI:** 10.1192/j.eurpsy.2021.441

**Published:** 2021-08-13

**Authors:** M. Tocco, A. Pikalov, L. Deng, R. Goldman

**Affiliations:** 1 Medical Affairs, Sunovion Pharmaceuticals Inc., Marlborough, United States of America; 2 Medical Affairs, Sunovion Pharmaceuticals Inc, Fort Lee, United States of America

**Keywords:** lurasidone, remission, schizophrénia, adolescence

## Abstract

**Introduction:**

Compared with adult onset, early onset schizophrenia is typically characterized by greater illness severity and less favorable prognosis.

**Objectives:**

To evaluate the proportion of adolescent patients with schizophrenia who achieved sustained remission and recovery during 2 years of treatment with lurasidone.

**Methods:**

Patients aged 13-17 years with a DSM-IV-TR diagnosis of schizophrenia, and a Positive and Negative Symptom Scale (PANSS) total score ≥70 and <120, were randomized to 6 weeks of double-blind (DB), fixed-dose treatment with lurasidone (37 or 74 mg/d) or placebo. Patients who completed 6 weeks of DB treatment were eligible to enroll in a 2-year, open-label (OL), flexible dose extension study of lurasidone (18.5-74 mg/d). Criteria for sustained remission, were the 6-month consensus criteria summarized by Andreasen (Am J Psych 2005;162:441-9). Criteria for sustained recovery consisted of meeting sustained remission criteria with a Children’s Global Assessment Scale (CGAS) score ≥70 for at least 6-months indicating no clinically significant functional impairment.

**Results:**

A total of 271 patients completed the 6-week DB study and entered the extension study, and 186 (68.6%) and 156 (57.6%) completed 52 weeks and 104 weeks of treatment, respectively. During OL treatment with lurasidone, 52.8% met sustained remission criteria, with a Kaplan-Meier (KM) estimate of 64.1 weeks for median time to sustained remission; and 28.8% met sustained recovery criteria, KM estimate of 104.6 weeks for median time to sustained recovery.

**Conclusions:**

For adolescents with schizophrenia, treatment with lurasidone was associated with high rates of sustained remission and sustained recovery over a two-year period.

**Disclosure:**

Employee of Sunovion Pharmaceuticals Inc. The study summarized in this Abstract was supported by funding from Sunovion Pharmaceuticals Inc

